# Various On-Chip Sensors with Microfluidics for Biological Applications

**DOI:** 10.3390/s140917008

**Published:** 2014-09-12

**Authors:** Hun Lee, Linfeng Xu, Domin Koh, Nikhila Nyayapathi, Kwang W. Oh

**Affiliations:** Department of Electrical Engineering, University at Buffalo, State University of New York (SUNY at Buffalo), Buffalo, NY 14260, USA; E-Mails: hlee24@buffalo.edu (H.L.); linfengx@buffalo.edu (L.X.); dominkoh@buffalo.edu (D.K.); nikhilan@buffalo.edu (N.N.)

**Keywords:** SPR, SERS, image sensor, smart phone, microfluidic chip

## Abstract

In this paper, we review recent advances in on-chip sensors integrated with microfluidics for biological applications. Since the 1990s, much research has concentrated on developing a sensing system using optical phenomena such as surface plasmon resonance (SPR) and surface-enhanced Raman scattering (SERS) to improve the sensitivity of the device. The sensing performance can be significantly enhanced with the use of microfluidic chips to provide effective liquid manipulation and greater flexibility. We describe an optical image sensor with a simpler platform for better performance over a larger field of view (FOV) and greater depth of field (DOF). As a new trend, we review consumer electronics such as smart phones, tablets, Google glasses, *etc*. which are being incorporated in point-of-care (POC) testing systems. In addition, we discuss in detail the current optical sensing system integrated with a microfluidic chip.

## 1. Introduction

Microfluidics has great potential to develop miniaturized systems for modern biology and chemistry by providing the ability to effectively control and measure small amounts of samples due to a need for high-throughput systems. In the conventional way in chemistry, most of the research has been performed in various glassware at milliliter scale for separation, reaction, and synthesis, which results in increased formation of products and lower yield, compared to a miniaturized system [1–4]. Therefore, the microfluidic approaches have been more attractive than their macroscale counterparts. Due to the advancements in manipulation of fluids in channels, with dimensions of tens to hundreds of micrometers, microfluidic systems, now more than ever, have the potential to provide a number of useful capabilities: the ability to manipulate reagent and sample volumes ranging from nano- to pico-liters range; to create higher degree of control for separations and detections; and to enable low cost and short time for analysis [5–7]. Therefore, the integration of a sensor in a microfluidic device can have great potential in developing total analysis systems for various applications such as the analysis of DNA, cells, and protein.

One of the most useful optical approaches consists of measuring refractive index changes by surface plasmon resonance (SPR) phenomenon [8]. Since the 1990s, optical sensors based on the SPR phenomenon have been seen as a useful technology thanks to their ability to detect sensitively variation in the refractive index caused by interactions on a thin metal surface [9]. SPR is an electromagnetic wave, propagating along the interface between dielectric and metallic materials by means of a charge density oscillation when satisfying a set of experimental conditions such as the thickness of metal film, the wavelength and the angle of an excitation light. An evanescent wave is also generated on the surface of dielectric material, linking the positively and negatively charged regions [10]. In general, the configuration of an SPR sensor consists of a glass prism coated with a metallic thin film [11]. When light is induced onto the metallic thin film, the charge density oscillation occurs by coupling between the excited light and the film, resulting in decreasing reflected light at a certain incident angle of the light.

Over the past few years, surface-enhanced Raman spectroscopy (SERS) has increased researchers' interest in developing sensing systems for molecular detection and recognition because of its high sensitivity. Intense signal amplification can be achieved when the molecules are immobilized on a metallic nanostructure. In addition, SERS reveals a spectral fingerprint for specific molecules since each molecule produces its own spectral signal [12,13]. SERS can be generated when light illuminates the nanostructure and the light is scattered from the nanostructures, which can be detected with an amplified intensity signal. Therefore, SERS can provide chemical and structural information on molecules without labeling target reagents [14]. This is one of the major reasons why there has been extensive research involving SERS in the fields of physical and analytical chemistry [15–18]. Another advantage of SERS is that structural information of molecules can be identified from a sample solution, which enables the SERS-based detection system to be useful in various analytical chemistry applications. The growth of nanotechnology has produced various nanostructures modified as probes to enhance the intensity signal and allow a highly sensitive response to small target materials [19]. As a result, the modification of nanostructures has been an important issue in SERS studies [10]. Recent progress has been made in designing an efficient system for transport of sample molecules to nanostructures and modifying the nanostructures to increase sensitivity thus realizing an optimized SERS-based detection system [20]. In this system, a simple microfluidic channel for a delivery of reagent is integrated on a flat silver surface and a 633 nm laser is illuminated through nanoholes on the silver surface which yields significant light amplification for SERS signal.

Optical imaging means using light to capture the data of an object's physical properties [21]. The ability to image nano/micro-sized structures and provide real-time and high-resolution physical information on samples makes optical imaging very useful in many biological applications (especially in diagnosis of diseases) and nanotechnology [22]. Although nano-scale imaging is very important in biology and nanotechnology, the conventional optical bright-field microscopy has limitations in terms of resolution and contrast because of the diffraction of light. Current techniques for overcoming these problems include electron microscopy, transmission electron microscopy (TEM) and scanning electron microscopy (SEM) [23]. Furthermore, to enhance image resolution, super-resolution optical techniques such as structured illumination microscopy and photo-activated localization microscopy (PALM) have been invented [24,25]. However, the conventional techniques suffer from complexity, high cost, and low throughput caused by small FOV and DOF. Hence, high throughput and the ability to detect nano-sized structure are desired. Additionally, recent academic interest has focused on developing more compact and miniaturized optical imaging devices for on-chip applications as this reduces the cost considerably and enables portability [26–30]. Currently, several approaches are being studied in order to achieve simple, compact, cost-efficient imaging sensors and overcome limitations in resolution, FOV and DOF. The in-line based lens-free integrated on-chip microscope [31–35] and on-chip fluorescent detection system [36–40] have proven to be promising approaches. Their ability to miniaturize a conventional optical system with maintained or improved performance is expected to have applications mainly in on-chip point-of-care or on-chip microscopic systems.

In the current age of consumer electronics (e.g., smart phones, tablets, Google glasses), new trends in point-of-care (POC) are expected to be based on a combination of microfluidics and consumer electronics for simplified readout, probably only electronic readouts, to eliminate the need for any imaging or optical equipment that would normally require advanced laboratory instruments. Driven by the massive numbers of mobile phone users, this fast evolving trend will change the way POC, including imaging, biosensing and diagnostics, is used [41].

Here, we review the recent progress made in terms of sensing technologies in microfluidic chips for various applications. First, we describe plasmonic-based technologies such as SPR and SERS integrated with microfluidic chips. Recent optical sensing technologies are reviewed for lensless microscopy and fluorescent detection system using CCD. Next, we discuss a new trend of sensing technologies based on the smart phone in POC. Finally, we describe a future trend of sensing technologies in biological applications.

## 2. Sensor Based on Plasmonics with Microfluidics

### 2.1. Surface Plasmon Resonance Sensor

**Figure 1.**
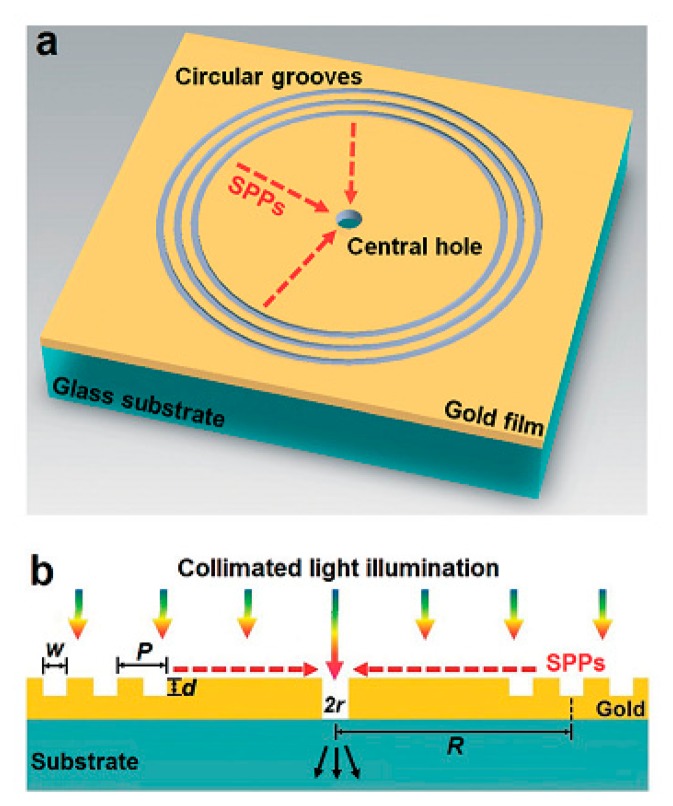
(**a**) Schematic of the interferometer. The interferometer consists of a 300 nm gold film with a nanoaperture surrounded by groove patterns onto a glass slide. (**b**) Side view and working principle. The groove patterns act as an efficient surface plasmonic wave coupler and then focus them to the central aperture (Reproduced with permission from [43]).

The new design concept based on SPR beyond the conventional Kretschmann configuration [42] is the transmission of collinear light beams to a specially designed geometry, which allows for simple optical components and cost-effective design, as shown in Figure 1 [43]. It is capable of performing massive multi-sensing for high-throughput systems. The capability can be significantly improved by integrating a microfluidic device in the sensing system because, in the microfluidic system, various liquid handling techniques have already been developed for a concentration gradient through the mixing of different reagents and can be easily adapted to the SPR sensor by means of collinear transmission. Many platforms considered previously, such as conventional LSPR and nanohole array sensors, pose a significant challenge because of broad spectral line widths, which can cause damping effect and absorption loss in a metal nanostructure [44–47]. Therefore, spectral signal shaping is critical for better performance of the sensors. In order to overcome the technical challenge, Gao and colleagues devised a novel approach based on optical interferometry for an array of circular shaped nanostructures [43]. The main advantage of the SPR system is that the spectral signal shape can be tuned by controlling the nanostructure to optimize the system. As a result, the sensitivity of the sensor is significantly increased up to 0.4 pg/mm^2^, which is comparable to a commercial SPR instrument (0.1 pg/ mm^2^). Additionally, a high FOM value of 146 was realized with the intensity detection method by varying the refractive index on the surface of the sensing area. The FOM is defined as (*dI/I*_0_)/*dn*. There is potential to increase the capability of sensitivity by tuning the sensing structure. Hence, an intensity-based sensing strategy for the interferometric device, based on SPR with CCD imaging technique to enable a miniaturized high-throughput system for point-of-care applications, has been realized.

**Figure 2.**
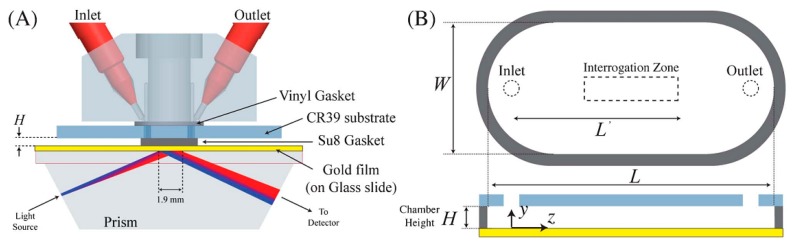
(**a**) The flow cells are fabricated on CR-39 substrate where 600 μm holes were drilled for a fluidic input and output. The top layer is then sealed by an SU-8 gasket to the sensing surface. The sensor chip is optically matched to a coupling prism with the gold-film. (**b**) Geometry of the sensing chamber for a delivery of ssDNA oligometers. Capture probes (biotinylated 20-mer ssDNA oligomers) are immobilized on the gold film which acts as the sensing surface (Reproduced with permission from [48]).

Practical understanding of reagent transport in a microfluidic channel is developed to determine how a biosensor system based on sensing surface reaction behaves by scaling the channel dimensions. Recently, the applicability of this scaling behavior was evidenced by decreasing the channel height for SPR sensor with the detection of ssDNA as shown in Figure 2 [48]. There have been efforts to find an optimized geometrical dimension of microfluidic channel on an affinity-based SPR sensor. Two parameters, channel aspect ratio (η = *L/H*) and mass transfer Peclet number structure, play a major role. The Peclet number is defined as Pe = *Q/WD*, where Q is volumetric flow rate, W is the width of the sensing chamber, and D is the diffusion coefficient of molecules. Both the experiment and the numerical simulation were conducted with the assumption Pe > η, and a scaling behavior of analyte flux (J) was demonstrated as $J\approx H^{-\frac{2}{3}}$ with constant volumetric flow rate and $J\approx H^{-\frac{1}{3}}$ with constant average fluid velocity. As a result, reducing the channel height from 47 μm to 7 μm significantly improved the sensitivity by a factor of four.

**Figure 3.**
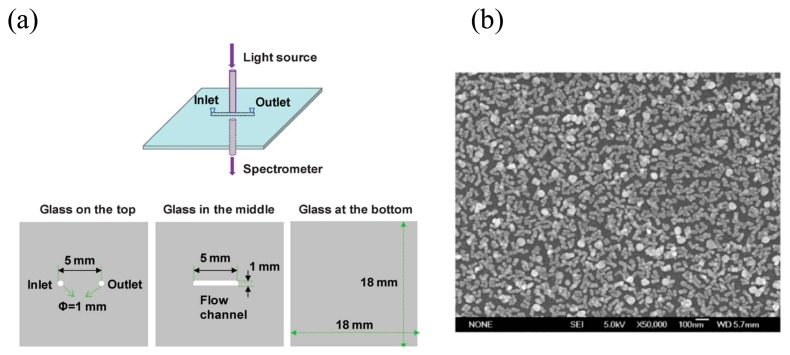
(**a**) Schematic of the LSPR sensing system with the microfluidic device fabricated on the glass substrate. The top image shows an assembled LSPR sensor chip and the bottom three glass slices with through holes and a fluidic channel. (**b**) Scanning electron microscopy (SEM) image of gold nanorods on the inner wall of the fluidic channel. The particle density is estimated to be ∼324 particles/μm^2^ based on 15 SEM images (Reproduced with permission from [59]).

Localized Surface Plasmon Resonance (LSPR) is another resonance phenomenon of waves localized in a metal nanostructure such as nanoparticles or nanowires that are small with respect to the wavelength of the optical wave [49]. Unlike an SPR based on a metal film, an LSPR of metal nanostructures has a distinction which means localized fields are tightly confined into the nanostructure, resulting in strongly enhanced fields [50–52]. The resonance condition of LSPR is also sensitive to the refractive index changes of the surrounding liquid medium on the sensing surface and the biological binding events on the metallic nanostructure [53]. The LSPR sensor has advantages as a detection system because of its high sensitivity and no pretreatment of sample for an enhanced detection is needed to allow fast qualitative discrimination of enantiomers. Gold nanoparticles and nanorods have been used as sensing material and the characteristics of gold nanorods were investigated by controlling the density of nanorods in a microchannel [54]. Recent work reported on LSPR sensors used single nanoparticle tracking to measure a peak shift based on a dark field microscope to allow for a multi-sensing platform. In addition, a normalization technique has been developed to avoid variations between particles in LSPR signals to realize a single nanoparticle sensor [55]. Generally, the LSPR sensor has been used to detect binding events [56], molecular reactions [57], and conformational changes [58]. Instead of chromatographic techniques that have typically been used for the analysis of chiral compounds, Guo and colleagues verified a biosensor integrated with microfluidic device using the LSPR to selectively recognize enantiomers of chiral compounds as shown in Figure 3 [59]. They achieved the first LSPR sensor on a microfluidic device where dense gold nanorods were formed on a self-assembled monolayer of 3-aminopropyltriethoxysilane (APTES) inside the microfluidic channel walls for promising drug–protein interactions. The preformed self-assembled monolayer on the surface of microfluidic channel allows for a simple approach to deposit the nanorods by attractive forces between them. The microfluidic channel was fabricated on a glass substrate where light passing through an optical fiber was induced into the channel for LSPR by a tungsten halogen light source and the LSPR signal was detected by a UV spectrometer. To form the gold nanorods on the surface of the channel, the self-assembled monolayers of APTES were immobilized. After that, the solution of gold nanorods was injected into the channel and incubated for five hours. The LSPR signal was detected by measuring the peak shift at resonance condition before and after binding events. As a result, a highly selective sensor was developed for an RS-melagatran with concentration ranging from 0.9 nM to 150 nM, whereas 10,000-fold amounts of SR-melagatran presented, which can interfere with the detection of RS-melagatran. The proposed method can be applied other enantiomers by modified receptors on the sensing material.

**Figure 4.**
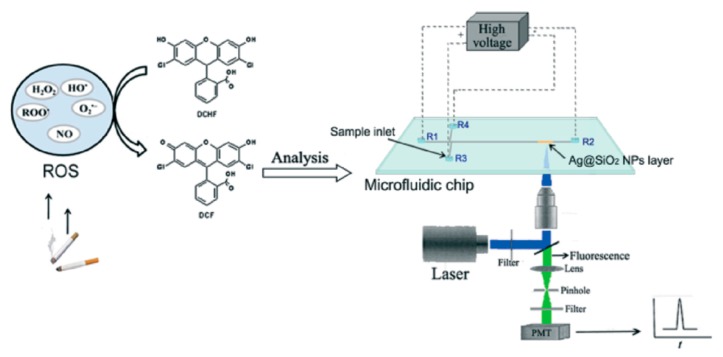
Schematic of the detection system with high sensitivity enhanced by LSPR. The DCHF are loaded into a sample inlet and the ROS is trapped from puffs of cigarette smoke mainstream. The generated DCF is quantified using the microfluidic electrophoresis system after the trapping process. A laser-induced fluorescence (LIF) detection system is used to detect fluorescent signal (Reproduced with permission from [60]).

Recently, one of the practical applications was demonstrated in the detection of reactive oxygen species (ROS) caused by cigarette smoke on an electrophoresis device using the LSPR as shown in Figure 4 [60].

Detecting ROS in cigarette smoke conventionally requires a large amount of cigarette smoke and takes tens of minutes. The integration of a microfluidic chip for electrophoresis in the sensing system can be useful for minimizing sample consumption and saving time. Sensitive determination of reactive oxygen species in cigarette smoke using microchip electrophoresis-localized surface plasmon resonance enhances fluorescence detection. The working principle is to measure a fluorescence intensity variation enhanced by LSPR when fluorophores get close to metallic nanoparticles which lead to changed spectral signals [61]. The sensitivity of the signal depends on the size, shape, and formation of nanoparticles [62]. Therefore, optimal sensitivity can be achieved thanks to nanoparticle properties. The electrophoresis device was used to quantify ROS by detecting dichlorofluorescein (DCF) produced by the reaction between dichlorodihydrofluorescein (DCHF) and ROS. Polyacrylonitrile (PAN) nanofibers coated with DCHF were used to facilitate the ROS trapping efficiency. A detection system integrated with an electrophoresis device increased the fluorescent signal threefold. A highly sensitive detection system was thus demonstrated and the limit of detection for DCF solution was 5.5 × 10^−11^M.

For better resolution in terms of microscopic techniques, a new technique called Surface Plasmon Resonance Imaging (SPRi) has evolved, which is used to quantify binding affinities and concentrations for various target molecules [63,64]. SPRi is a high-throughput optical detection approach similar to the SPR in principle, and senses multiple interaction spots at once by using a parallel detector such as a CCD camera [65]. Because of a refractive index change on the sensing surface caused by binding events, reflected light intensity is changed, resulting in different intensity of reflected light at sensing spots. Observing change in the intensity of the reflected light through an image captured by a CCD camera permits spatial monitoring of binding events.

**Figure 5.**
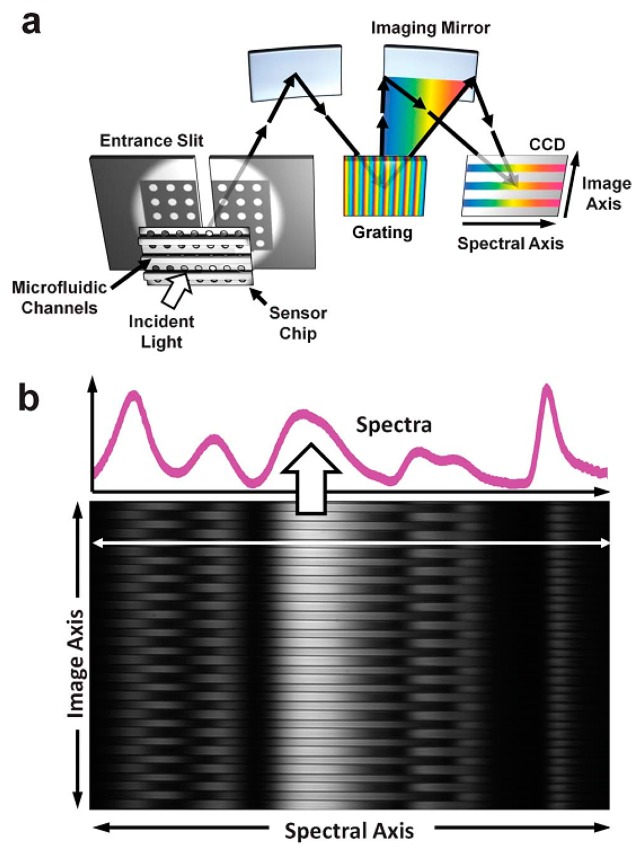
(**a**) Schematic of SPR imaging setup using nanohole arrays and parallel microfluidic channels. (**b**) Sample date measured by a captured CCD image. Spectral signal along a horizontal line on the sensing area shows the transmission spectrum for a single microfluidic channel (Reproduced with permission from [68]).

Recently, SPR imaging has been used as an attractive sensing platform for simultaneous multi-reagent recognition and quantification of binding events because of the demand for a high-throughput system [65–67]. Despite the promising SPR phenomenon in conventional configurations, the challenge of multiple reagent or ligand detection remains since the SPR signal measurement should be repeated, which can be a labor-intensive process [68]. Generally, microarrays are functionalized to capture reagents or ligands on metallic sensing surfaces. For example, a sample solution can be introduced into the microarray to allow biological binding events on the sensing surface in which light is illuminated to excite the SPR. The SPR signal is very sensitive to refractive index changes caused by binding events on the microarray. The reflected light intensity from the microarray will be decreased as much as the excitation of SPR, resulting in different spatial intensity. Thus, the captured images of the sensing surface can be analyzed to monitor the surface binding events in real time, which allows for highly sensitive detection systems [69]. Intensity-based SPR imaging recognition has been widely used as microarrays can have very high density of sensing spots, which enable high-throughput experiments. Recent advances in SPR imaging systems have been described by Lee and colleagues, who report full spectral imaging with 50 microfluidic channels capable of analyzing multiple detections on large nanohole arrays [68]. As shown in Figure 5, the microfluidic channels are bonded to the sensor, and then the tungsten halogen lamp is shone on the sensing surface to excite SPR. The transmitted light is introduced into a 50 μm wide slit and spectrally dispersed by a grating. The reflected light from the grating and imaging mirror is recorded on the CCD camera in real time. The spectral resolution of the proposed SPR imaging system is 7.7 × 10^−6^ RIU. As a result, spectral images are highly uniform over all channels which isolate sample solutions without cross-contamination. As proof of concept, binding reaction between cholera toxin b subunit (CTX-b) and ganglioside (GM1) was performed and the calculated limit of detection was 8.4 nM, which is comparable to the minimum concentration of other spectral peak shift detection systems.

**Figure 6.**
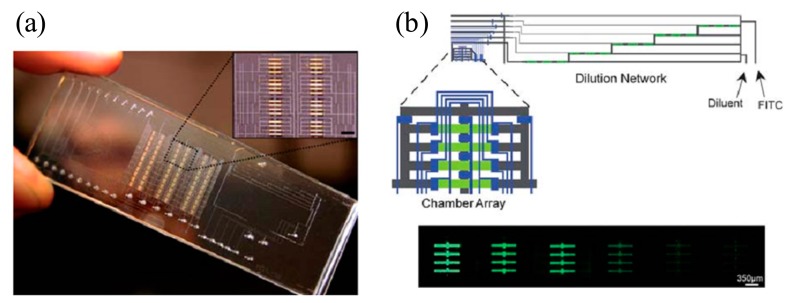
(**a**) Real image of the microfluidic device on a glass slide with gold patterns as a sensing surface. The inset shows a close-up region of the microarrays. (**b**) Top: Dilution channel network for a chaotic advection micromixer between 45 mM fluorescein and ddH_2_O, Bottom: image of fluorescent intensity to show six different concentrations of the fluorescein generated by the dilution network (Reproduced with permission from [65]).

Current commercial instruments lack multiple detections because they have only a single fluid flow channel. One of the main advantages of the SPR imaging technique is its ability to detect various reagents or ligands simultaneously with high sensitivity [70]. To avoid time-consuming and labor-intensive experiments in binding events, an SPR imaging system with a microfluidic array has been used to generate output signals in a high throughput fashion [71–73]. The integrated microfluidic device will allow for manipulation of fluid effectively and quickly to create a concentration gradient on a sensing surface. To address this concern, a microfluidic array was integrated with an SPR imaging system by Ouellet and colleagues, who reported that a device consisting of 264 chambers with 700 pL reaction volume isolated by 1132 microvalves is capable of investigating 264 different samples in a single experiment, as shown in Figure 6 [65]. This microfluidic device is used to generate six different concentrations via a serial dilution channel network. The valves integrated in the microfluidic device consist of two layers; the top layer is a flow channel and the bottom one is a control channel to deflect thin membranes in both upward and downward directions pneumatically. The system showed detection of human α-thrombin on the sensing surface immobilized with anti-human α -thrombin IgG. The sensing surface of the array of 25 × 25 mm and 12.6 μL/h was used as a volumetric flow rate to decrease reagent consumption. The serial dilution network was designed and tested with 45 mM fluorescein for the different concentrations where a 1:1 mixing ratio was successfully obtained at each junction, resulting in relative standard deviation of 0.092. Dissociation constant (*K_d_*) equal to 5.0 ±1.9 nM was achieved and the value is comparable to a commercial product.

**Figure 7.**
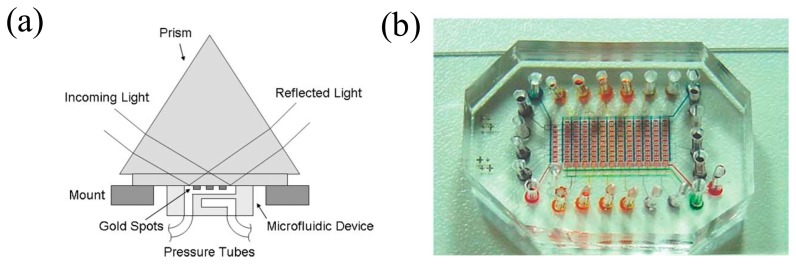
(**a**) The conventional configuration for SPR imaging system. A collimated laser beam (λ = 625nm) is passed through a prism and illuminated on the gold spots. The reflected beam is collected by a digital imaging device. All binding events on the gold spot in the microfluidic channel are monitored in real-time. (**b**) Image of the microfluidic device in which valves and channels are integrated. To visualize the valves and channels, a food color dye is injected (Reproduced with permission from [67]).

Luo and colleagues used SPR imaging techniques to develop a microscale platform for binding events as shown in Figure 7 [67]. The binding events between antibody and antigen were monitored on an SPR imaging setup for about 10 min. The microfluidic device integrated consisted of two layers to control a sample reagent as described above. The two sets of channels fabricated were 100 μm wide and ∼10 μm high, to allow four sets of the same experiment for the immunoassay. As a sensing material, a circular-shaped gold pattern array was used with a diameter of 250 μm and a thickness of ∼50 μm which was exposed to sample reagents for binding reactions. All liquid flows were pumped by pressure generated by nitrogen gas. For SPR imaging, the prism-based Kretschmann configuration was employed and an HeNe laser (λ = 625 nm) p-polarized was shone on the sensing surface. Afterwards, the reflected laser beam was detected by a digital imaging device. To test the binding events between biotin-BSA and anti-biotin Ab a single-step binding was realized on the setup in 10 min and the performance of the system with a sub-nanomolar detection limit was demonstrated. In two-step binding, a signal amplification was achieved by using gold nanoparticles conjugated with anti-goat IgG antibody. As a result, the enhanced limit of detection was brought down to ∼40 pM.

### 2.2. Surface-Enhanced Raman Scattering Sensor

**Figure 8.**
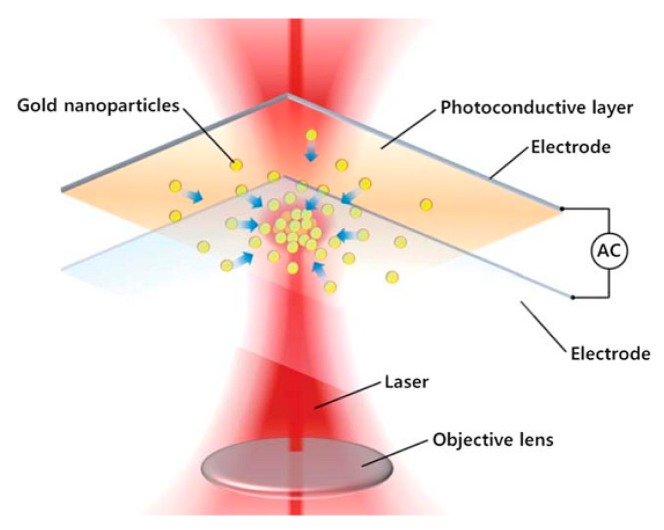
Schematic of the OET-based SERS sensing system, allowing on-demand generation of SERS-active sites and *in situ* measurement of the enhanced SERS signal (Reproduced with permission from [78]).

There are two conventional methods for combining SERS systems with a microfluidic device: one is to manipulate nanostructures in liquid samples, and the other is to define the SERS sensing region by using nanostructures [74]. Despite its huge potential, it is difficult to achieve uniform mixing of samples through a sensing region. To overcome the technical challenge, research has focused on microfluidic platforms which can enable uniform mixing of samples and form well-controlled aggregation of nanostructures to detect target samples [75]. A droplet-based microfluidic device has also been used for online SERS signal detection for high throughput systems [76]. An important SERS advance recently reported on-demand creation of SERS sensing spots using a microfluidic device where an alternating current was applied to allow metallic nanostructures to be concentrated for dynamic and highly sensitive spectral signal detection [77]. In the dynamic and active SERS platform, the aggregation of metal nanoparticles can be easily controlled, which provides the high sensitivity since the nanoparticles act as the SERS signal enhancers. For the platform, a conventional SERS sensing system is used and the microfluidic device is fabricated with a glass slide coated by a photoconductive layer which can produce virtual electrode patterns on the region of interest, as shown in Figure 8. Only a single 5 mW HeNe laser source was used both to create the SERS signal and to concentrate nanostructures at the region of interest with a small amount of sample volume. The working principle is to manipulate particles and fluids with an optically induced electric field [78]. The most common method for electrokinetic motion is an optoelectronic tweezer (OET) device constructed with a photoconductive layer on a plate electrode. When a laser beam illuminates the photoconductive layer, the current can be generated creating a non-uniform electric field in a chamber to control the particles or fluids. The non-uniform electric field can exert forces to concentrate particles or fluids [79,80]. The frequency of the applied electric field was investigated from 100 kHz to 100 Hz with fluorescence intensity of rhodamine 6G. It was found that the fluorescent intensity of rhodamine 6G decreased with decreasing frequency. Although the mechanism of this phenomenon is not clear, the intensity change was not significant at 100 kHz. When the gold nanoparticles were concentrated in the sensing region by applying 20 V_pp_ at 100 kHz, an increased SERS signal was observed. For practical applications, a target sample of 250 μM adenine was detected on the SERS system. With a well-controlled active SERS system, the highly sensitive detection system was demonstrated successfully. It requires a small amount of sample volume (∼500 nL) without operating components such as a microvalve or micropump.

**Figure 9.**
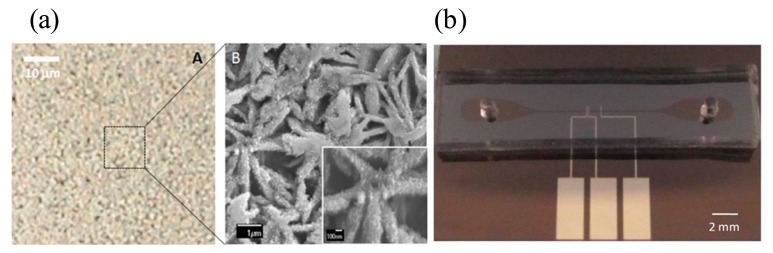
(**a**) The image of silver nanoparticles decorated on the copper-core/carbon-sheath nanowalls (**b**) The image of microfluidic device with three electrodes (counter, working, and reference electrodes that are ordered from left to right) to synthesize the nanostructures on the working electrode by the electrodeposition method. A constant potential of − 2.7 V is applied to the working electrode against reference electrode for 60 s. The counter electrode is used to allow a current to flow (Reproduced with permission from [81]).

Recently, new silver nanoparticles on nanowalls were synthesized in a microfluidic channel to enhance the sensitivity of the SERS signal significantly, as shown in Figure 9 [81]. To fabricate the silver nanoparticles-deposited nanowall structures, an electrodeposition method and silver galvanic replacement reaction method were used for copper-core/carbon-sheath nanowalls and for silver nanoparticles, respectively. The sensitivity was affected by several factors: (1) large surface area of silver particles on nanowalls for effective absorption; (2) many nanocavities caused by nanowalls for surface plasmon coupling with the incident laser; and (3) “hot spot” chemical effect [81,82]. To verify the performance of the novel nanostructure for the SERS signal, crystal violet was employed as a model compound and an Ag-ion laser illuminated the sample. The enhancement factor for the SERS signal in the given system was calculated to be 1.1 ×109. The factor is given by the following equation:

$${\text{EF}}_{\text{app}}=\frac{I_{\text{SERS}}/C_{\text{SERS}}}{I_{NR}/C_{NR}}$$

where, *I_SERS_* and *I_NR_* are the intensity of SERS and the intensity on a substrate, respectively and C_SERS_ and C_NR_ are the concentration of molecules contributing these intensities, respectively. As a result, an ultra-sensitive SERS-based system was achieved successfully with the microfluidic device.

**Figure 10.**
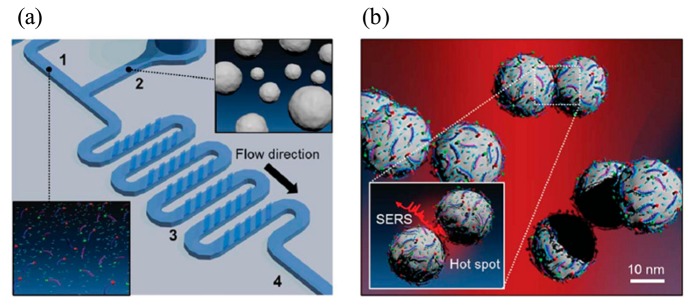
(**a**) Microfluidic channel for mixing of (1) DNA oligomer mixtures and (2) silver nanoparticles. The (3) groove structure is designed to increase the efficiency of the mixing on the microfluidic channel and SERS signal is detected in the (4) downstream region (**b**) SERS signal coming from DNA oligomer on silver nanoparticles when hot spots are generated between them (Reproduced with permission from [83]).

A microfluidic device for generating a concentration gradient with gold nanospheres and microarray wells demonstrated advanced progress in terms of the SERS sensing system, as shown in Figure 10. It can produce a simple and reproducible platform for cancer biomarkers in a high throughput fashion without a manual dilution process. For proof of concept, an alpha-fetoprotein (AFP) marker was detected quantitatively. In addition, the total analysis time was under 60 min and the limit of detection of 1 ng/mL was obtained for rabbit AFP antigen on the proposed SERS based system [83]. Another important application for SERS was reported by Choi and colleagues, who demonstrated a very useful technique for two different types of DNA oligomer at the same time [84]. In DNA analysis, polymerase chain Reaction (PCR) is typically required to amplify the DNA because of very low concentration. To detect the amplified DNA samples, an optical method such as fluorescent or chemiluminescent detection has been used. However, the SERS system developed does not need any amplification and enables the detection of various DNA oligomer mixtures simultaneously. Therefore, the system has great potential for use as an analytical tool for detection of multiplex DNA.

## 3. On-Chip Optical Image Sensor

### 3.1. In-Line Holography Based on Optical Lensless Microscopy

Removing the lens in microscopy, and then using holography and pixel super-resolution to construct images could achieve a simpler platform with better performance over larger FOV and DOF. Limitations in diffraction and resolution are the greatest challenge concerning performance of conventional optical microscopy and could be overcome with a lensless optical imaging technique, holography and pixel super-resolution. Holography is constructing image using scattering and interference of light and pixel super-resolution is the technique of enhancing optical image which has limitation in resolution (low resolution images) by combining multiple pictures.

**Figure 11.**
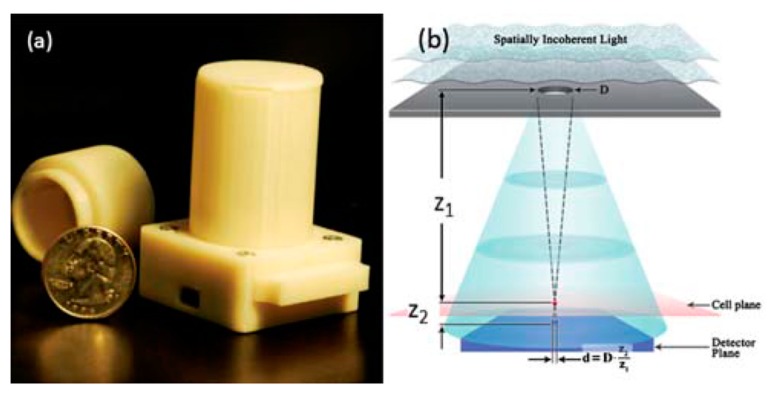
(**a**) A lensless on-chip microscope based on holography powered by USB connection which utilized LED sources and CMOS sensor array. (**b**) Schematics of the microscopy. The size of the hole is 50 to 100 μm and it is 3 to 4 cm above the sample. This allowed to cover FOV of ∼24 mm^2^ (Reproduced with permission from [31]).

Illumination of incoherent or partially coherent light is scattered by the presence of a sample, and unscattered light is detected by sensory array. With the received optical information, hologram images are constructed by converting phase information to intensity oscillation. Multiple hologram images are taken and merged together to improve the image of the sample. This approach could provide an image of a sub-100 nm sized nanoparticle or virus with a large FOV, >20 mm^2^, and simple platform [31]. Furthermore, the integration of a microfluidic channel could provide real-time detection. In 2010, Mudanyali and colleagues demonstrated a lens-free on-chip imaging device as shown in Figure 11 for telemedicine application. The device weighed 46 g with dimensions of 4.2 cm × 4.2 cm × 5.8 cm and could achieve 1–2 μm resolution (about a 10-fold improvement compared with a lens imaging device). First, LED was used as a source of incoherent light and the light was initially filtered to pass through a large pinhole of 50–100 μm diameter. Use of LED as light source could suppress coherent speckle noise and additionally, the hole had eliminated the need of coupling and focusing of light [31]. Then, the light was propagated in air over a distance of 3 to 4 cm and interacted with the sample. The presence of the sample induced scattering, absorption and refraction of light depending on the physical property of the sample (for example, size, 3D morphology and refractive index). The interference of the light waves had further propagated through the sample with unscattered light propagated and created a hologram of the sample by CMOS sensor array.

The device was able to provide images of various cells and particles such as blood cells, platelets, focused-ion beam (FIB) fabricated objects, and 3, 7 and 10 μm polystyrene particles. Furthermore, the hologram image could be reconstructed via differential interference contrast microscopy (DIC) to provide a clearer image of the sample. In DIC microscopy, a thin birefringent crystal was placed between two cross-polarizers and two waves were generated which were spaced 1.1 μm and polarized orthogonally. Two waves had generated two holograms which consequently creating a new hologram as they were interfere at the sensor plane. Consequently, a new hologram is generated. Its FOV was 24 mm^2^ when bench-top optical microscopes had an FOV of 4 mm^2^ with a 10× objective lens; therefore a new platform could capture an image of larger volume. Moreover, it was a lensless platform which used a CMOS imager chip for a simpler and more cost-effective approach in biomedical sensing [32]. A digital hologram of cells provides a simple cell signature which varies slowly with time and position and is very useful in cell tracking. A demonstration of the tracking motion of 3T3 cells over 20 h on ECM-coated hydrogel plate was performed (sampling period was one minute) in high temperature and humidity conditions [32]. It was shown that more stiffness causes more motility of cells and the death of cells causes loss of tracking. Because of the short distance between the sample and light source (pinhole or aperture), the device could achieve greater FOV and throughput and was simpler and cheaper to make. Moreover, the shorter sample-to-sensor distance increased the light collection efficiency and allowed sub-pixel resolution imaging.

**Figure 12.**
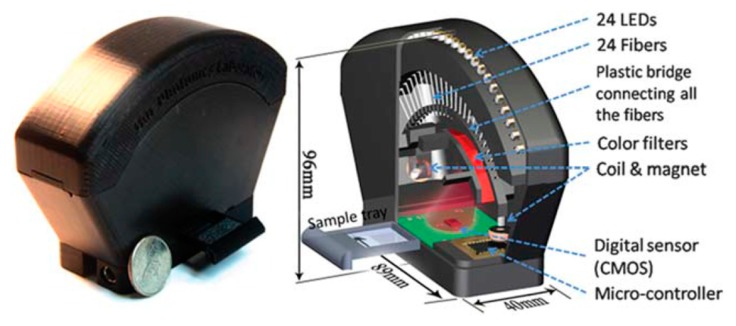
An image (**left**) and a schematic image (**right**) of the field-portable lensless tomographic microscope. It weight ∼110 g which employed 24 LEDs as light source and the LEDs were mounted along arc structure for providing images at different angle (∼50°). It could achieve resolution of < 7 μm which can cover ∼20 mm^2^ (Reproduced with permission from [33]).

Another type of on-chip Lens-free microscope, a portable lensless tomographic microscope device, was developed by Isikman and colleagues, as shown in Figure 12 [33]. The device can achieve comparably enhanced 3D resolution, sub-micron resolution (<7 μm) with an FOV of 20 mm^2^ and DOF of 1 mm. Because the device employs varying illumination angles for tomographic imaging (allowing imaging sections of sample by different angle of illumination), it is possible to capture multiple digital in-line holograms of the sample and achieve electromagnetic actuation for pixel super-resolution to improve the lateral resolution of lensless holograms. In the experiment, 24 LEDs of coherent light source were used and each LED was coupled with an array of optical fibers (the diameter of each optical fiber was 0.1 mm and thus no focusing lens was required). The optical fibers were connected to a plastic bridge with neodymium magnets at both ends and electro-coils were mounted to shift the optical fibers along both x and y directions by electromagnetic actuation of magnets. As the fibers were shifted to a new position, new 2D holograms were captured by the sensor array under the sample tray. At different fiber angles, multiple images were taken and digitally reconstructed to create projection images of the samples. Then the images were merged together to compute tomograms of the samples. The device has high-throughput with long DOV (1–4 mm) and wide FOV (20 mm^2^) and it can reconstruct holographic projection at any depth; additionally, tomograms can be computed without introducing spatial errors. Hence 3D imaging of any area within the DOV was achieved, demonstrated by imaging a multilayer chamber (separated by 1 mm) composed of 10 μm size beads and imaging a 40 μm *Hymenolepis nana* egg. In order to do pixel super-resolution tomographic imaging of thick layers, extra signal processing was required and in the demonstration a newly developed algorithm was used to calculate the given depth of the layer. Consequently, the device provided images of samples at different depths. The results of demonstration showed that the device could achieve large FOV (by placing a LED far from the sample) and increase spatial resolution without reducing FOV. Moreover, pixel super-resolution and computed tomography improved the lateral resolution of lensless microscopy significantly. Also, the possibility of integrating the device with a microfluidic platform which carries samples into the device could enable real-time imaging and detection (2D images could be taken as the samples flow within the microfluidic channel and reconstruct high-resolution holograms).

**Figure 13.**
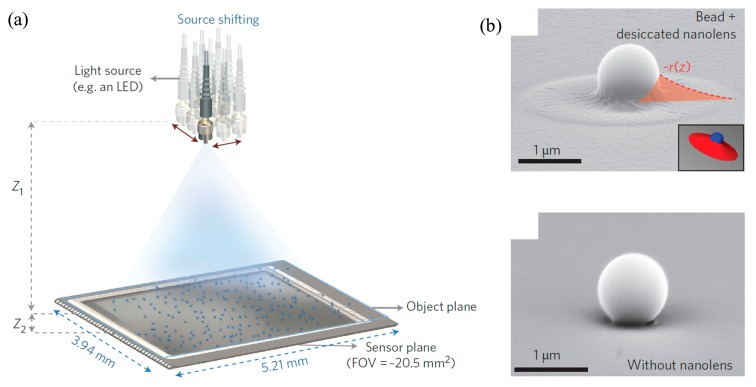
(**a**) Schematic image of on-chip lensless microscopy with nanolenses. The distance from light source to sample is 8–12 cm and the light source can move 300 μm along the plane which could cover area of ∼20.5 mm^2^ (**b**) SEM image of nanolenses with (top) and without (bottom) nanolense (Reproduced with permission from [34]).

Furthermore, a new approach for achieving large FOV lens-free on-chip microscopy by employing self-assembled nanolenses as shown in Figure 13 was developed for wide-field nanoparticle imaging. Implementing self-assembled nanolenses in nanoparticle imaging could achieve compact, cost-efficient and high-throughput platforms and detection of sub-100 nm nanoparticles across FOV of > 20 mm^2^ by improving SNR and contrast over large FOV (not by enhancing spatial resolution). Integration of two techniques, self-assembled liquid nanolenses and on-chip lensless computational microscopy, is the key to progress. In spite of using nanolenses, the overall concept of the device relied on lensless on-chip microscopes because the nanolenses were not for focusing light but they were installed on each pixel of the sensor array. Nanolenses are biocompatible buffers and are mainly used for spatial phase masking to improve lensless diffraction and prevent aggregation of nanoparticles [34]. Before the use of liquid nanolenses, the effect of wetting film on the performance of lensless on-chip microscopes was studied and demonstration proved that wetting film enhances SNR and contrast significantly because wet-thin film could reduce noise. In this system, nanoparticles were mixed with buffer solution, 0.1 M Tris-HCl and 19% PEG 600, then nanolense were formed around sample particles by applying mechanical vibration for creating and the performance of the approach was tested by improced imaged of improved images of *E. coli, Giardia lamblia* trophozoites and human RBCs [35]. The same buffer was used for liquid nanolenses, but treated with plasma for creating nanolenses around sample particles, with pixel super-resolution (implemented by source shifting) in a later demonstration. A quasi-monochromatic fiber-coupled light source was used to illuminate the sample 8 to 12 cm away from the plate and pixel super-resolution was achieved by shifting the position of the light source. This allowed imaging of the H1N1 virus and sub-100 nm adenovirus which had greater SNR and contrast than ×100 oil-immersion objective lens microscopy over 20.5 mm^2^ FOV. Lensless optical microscopy based on holography is expected to be applied in telemedicine since it is cost-efficient, simple, and gives better performance over a larger volume. Nevertheless, it suffers from high SNR because of weak scattering of sub-micron cells. Also, it has low selectivity which means it cannot be directly used in on-chip fluorescence imaging.

### 3.2. CCD Sensor for Fluorescent Detection

**Figure 14.**
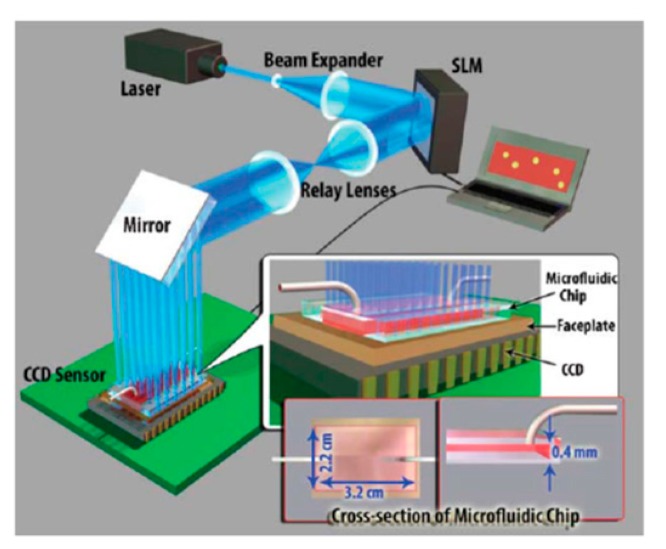
Schematic image of high-throughput on-chip fluorescent detection platform. CCD, structured illumination array is integrated together for generating image of fluorescent sample in microfluidic chip size of ∼22 × 32 × 0.4 mm. This could capture 144 lensfree images within 36s (Reproduced with permission from [37]).

An on-chip fluorescent detection device enabling high-throughput imaging of whole blood by structured illumination, as shown in Figure 14, was developed by Arpali and colleagues [37]. A whole-blood sample was injected into a microfluidic chip (maximum volume 0.3 to 0.7 mL), on an optoelectronic sensory array which was then scanned by a digital spatial light modulator (generating a 2D array of Gaussian excitation spots). A lens-free fluorescent image of the sample was captured at each spot and as a result a sequence of 144 images was constructed from the same sample (the images were merged digitally to enhance the SNR and contrast to construct better image). The approach was demonstrated by imaging an undiluted whole-blood sample. A large microfluidic channel (22 mm × 32 mm ×0.4 mm) was placed on an optical fiber faceplate [38] (composed of many optical fibers) coupled to a sensor array (CMOS or CCD) and fluorescent dyed samples in the channel were excited through structure illumination setup (light generated by SLM). The structured illumination used lenses to cover the large area and SLM generated 28 × 21 Gaussian spots. Then, 36 × 36 SLM pixels were assigned to each Gaussian spot for imaging (the size of each pixel was 19 μm). As a result, both the structure illumination setup and sensory array could cover a large imaging area of 7 to 18 cm^2^ [31]. To evaluate the approach, two different images, each using different illumination systems, were taken. One image was taken by plane wave illumination and another by structured illumination and they were compared. The images taken by structured illumination showed hidden fluorescent dyes at flow rates ranging from 50 particles/mL to 1500 particles/mL. The result showed that the fluorescent detection platform with a structured illumination system could achieve more sensitive and accurate detection at high flow rates even with whole-blood samples. In conventional fluorescent detection, a large volume of whole blood cannot be used (it is limited to less than 0.2 to 0.3 μL) because of the high-scattering and absorbent nature of whole blood and the limitations of DOV and FOV. Therefore this new approach with larger volume accessibility is a good solution to the current challenges in fluorescent detection.

**Figure 15.**
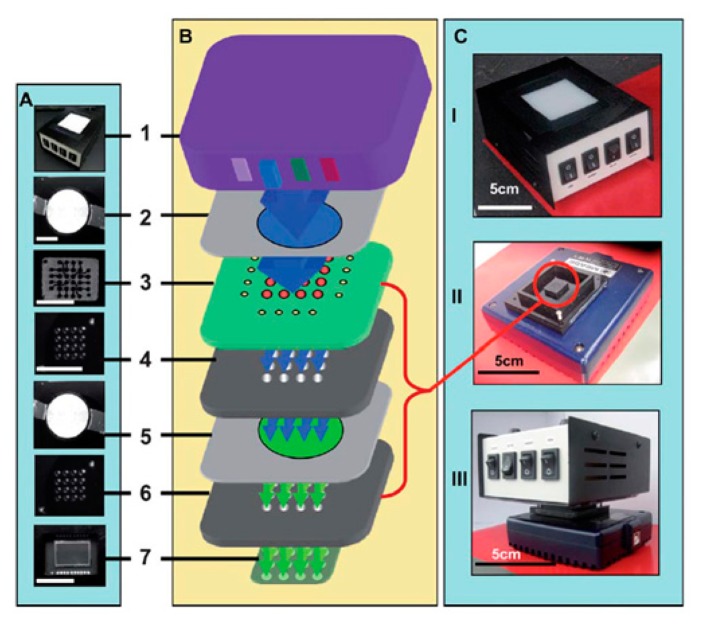
(**A**) Photograph of elements of on-chip fluorescent detection platform using Söller collimators. (**B**) Schematic image of elements of device. The image 1 is the main element which contained multi-wavelength LEDs. Image 2 and 5 are filters (blue and green emission filter respectively), and image 4 and 6 are light collimators. The microfluidic channels were shown in image 3 and 7 is the CCD camera. (**C**) Photograph of assembled device (Reproduced with permission from [40]).

A new fluorescent detection mechanism was developed by Balsam and colleagues using micromachined optical Söller collimators with a CCD sensor array, as shown in Figure 15 [40]. A collimator is an optical element which can be used in uniform spatial distribution of light and it filters a beam of parallel rays by allowing light to pass in a specific direction; as a result, the blurriness of the image is removed. A Söller collimator is a sheet of acrylic opaque material with tiny holes which allow rays parallel to the hole and is used for aligning incoming light. Using a Söller collimator instead of a lens allowed filtering of fluorescent emission (by filter) by imaging the decoupling of the sensor array and the surface, and enabled separation of the microfluidic channel from the sensor array. Consequently, fluorescent detection can be achieved by using a simple, low-cost, low-power, compact and portable but multifunctional device.

As described in Figure 15, illumination of RGB LEDs excited fluorescent dye in a 16-well-like microfluidic device after passing through an excitation filter. The emitted fluorescent light traveled through two stages of Söller collimator (removing unwanted rays which can create blurry images) and was recorded by a CCD sensor array [33]. Demonstration of the device was done through detection of botulinum toxic activity by means of a FRET-based peptide assay for observing activity of the BoTN-A light chain. A sample of 10 μM BoTN-A bound with FITC fluorescent was prepared and diluted for demonstration (from 0.0195 to 20 nM). A minimum of 1.25 nM could be detected. With a Söller collimator, the use of an interchangeable filter and sample were enabled because of the separation of the CCD and microfluidic chip; additionally, the emitted fluorescent light was directed towards the CCD, which minimized undesirable scattering of light. A low-cost and simple platform with high selectivity and sensitivity as well as the ability to detect large volume is one of the advantages of the on-chip fluorescent detection system. However, the need for sample preparation increases the operating time and some approaches, such as Söller collimator-integrated fluorescence detection, require more time in order to muster sufficient intensity for detection and need to manage background noise carefully.

## 4. Imaging, Biosensing and Diagnostics Based on Smart Phone Platform

### 4.1. Microscope Based on Smart Phone

**Figure 16.**
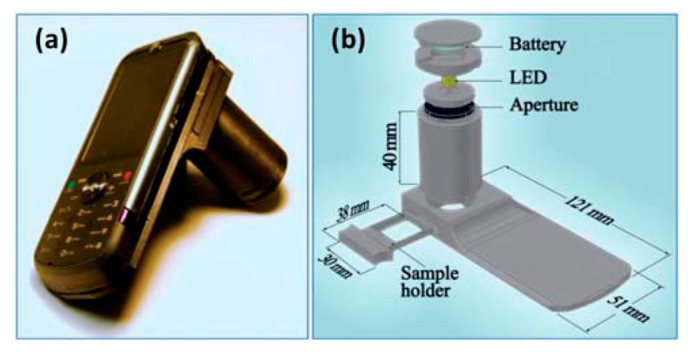
(**a**) A lensfree cellphone microscope which operates based on incoherent in-line holography is shown. The additional hardware installed on the cellphone weighs ∼38 g (<1.4 ounces) and is composed of an inexpensive light emitting diode (at 587 nm) with an aperture of ∼100 mm in front of the source. This cellphone microscope does not utilize any lenses or other bulky optical components and operates with a unit fringe magnification to claim the entire active area of the sensor as its imaging field of view. The samples to be imaged are loaded from the side through a mechanical sample holder. (**b**) Schematic diagram of the microscope attachment shown in (a) is illustrated (Reproduced with permission from [85]).

Tseng and colleagues designed a customized add-on component which can be attached to the camera of the cellphone, as illustrated by Figure 16 [85]. The aim of this study was to realize lens-free digital microscopy with a cellphone by means of partially coherent digital in-line holography and to offer a cost-effective tool for telemedicine applications without any lenses, lasers or other bulky optical components. As the LED source is far away from the sample plate (∼4 cm) and the relatively large size of the aperture (∼100 μm), it means that partially coherent holography of the sample is generated and recorded by the cellphone. Based on the image processing algorithms, microscopic images can be reconstructed from diffraction patterns. Therefore, by taking holograph pictures and image processing software, they successfully imaged variously sized micro-particles, as well as micro-sized cells and bacteria. They reported a lens-free microscopy with a sub-pixel resolution of ∼1.5–2 μm on a cellphone camera with ∼2.2 μm pixel size at the CMOS sensor. Moreover, the reconstruction of the microscopic image can be done in a central hospital with powerful GPU. Therefore, the end-users only need to take pictures with their cellphones and send them to the hospital for image processing and analysis. Because of the restricted size of the camera pixels, the resolution of the reconstructed microscopy images is limited.

**Figure 17.**
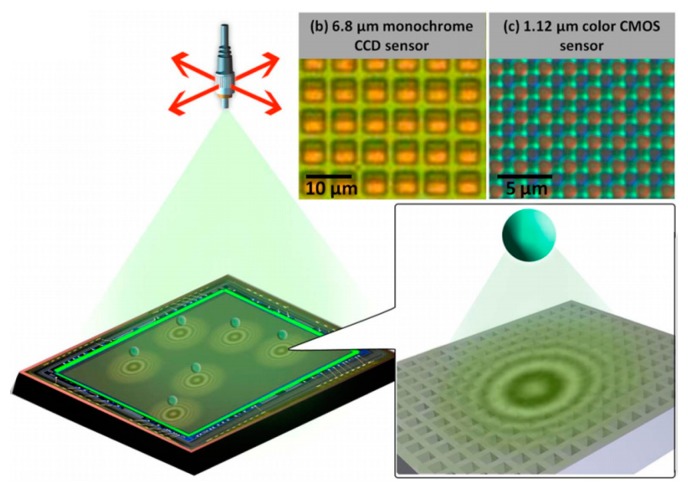
Lensfree on-chip microscopy setup. (**a**) Schematic of the lensfree holographic microscopy setup. The close-up of (a) shows that the scattered wave from the object interferes with the unperturbed reference wave and forms an in-line hologram, which is then sampled by the image sensor chip. The pixel structures exhibit large variability in terms of pixel pitch and morphology as can be seen in (b) and (c). (**b**) Shows an optical microscope image (20× objective, NA = 0.5) of a 6.8 mm monochrome CCD image sensor chip. (**c**) Shows an optical microscope image (100× Water immersion objective, NA = 1) of a 1.12 μm color CMOS image sensor chip, where the Bayer pattern can be readily seen (Reproduced with permission from [86]).

As mentioned previously, because of the pixel-size limitation of lens-free on-chip microscopy, the spatial resolution is restricted. Greenbaum and colleagues proposed a mechanism to circumvent this restriction by employing pixel-super-resolution techniques to synthesize a smaller effective pixel compared with the actual one [86]. Pixel-super-resolution techniques are based on computational methods to overcome undersampling of an image owed to pixel-size limitation by generating a high-resolution image from a series of lower-resolution images of the same objects taken by sub-pixel shifts of the LED light source (Figure 17). They achieved an effective pixel count of 2.52 billion with a monochrome CCD image sensor of 6.8 μm pitch and obtained an effective pixel count of 1.64 billion with a color CMOS image sensor of 1.12-μm pitch. The combination of pixel super-resolution and hologram de-convolution enabled an NA improvement factor of ∼3 to be realized. They demonstrated that by employing a short wavelength LED (=372 nm), their proposed method can resolve periodic grating lines with a line width of 225 nm and can capture wide-field on-chip imaging of multi-walled carbon nanotubes with a diameter of ∼160 nm based on two different sensor arrays that are very different in terms of pixel size, circuit architecture and readout mechanisms. Though a higher resolution is achieved compared with previous lens-free on-chip microscopy methods, the control of sub-pixel shifts is not easy and very hard to integrate in a compact setup.

**Figure 18.**
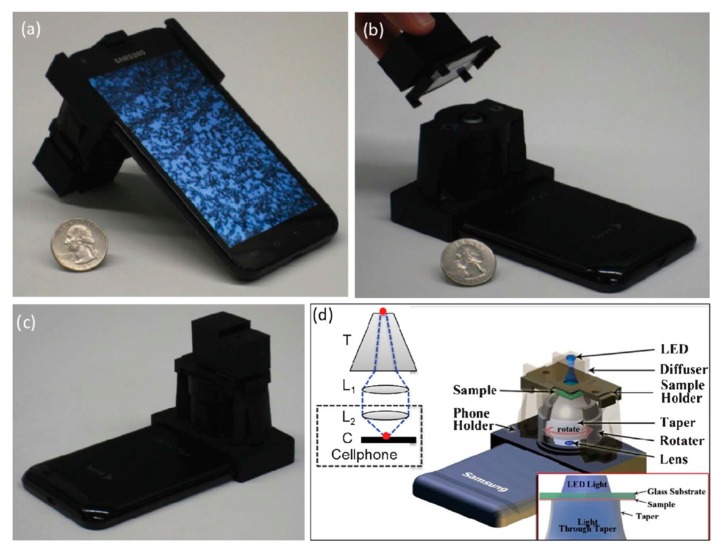
(**a**–**c**) Photographs of the Contact Scope installed on an Android cellphone from different views are shown. (**d**) Schematic diagram of the cellphone attachment of the Contact Scope is demonstrated, where the planar samples of interest are positioned in contact with the top facet of a tapered fiber-optic array (*i.e*., denoted as taper) and are illuminated by an incoherent light source (e.g., a simple LED). The transmission patterns of the micro-objects are magnified by the taper (T) and then imaged onto the cellphone CMOS chip (C) through an additional lens (L_1_) and the built-in cellphone lens (L_2_). Keeping the sample at a fixed position, the taper is rotated with discrete angular increments of e.g., 1–2 degrees, creating a sequence of raw contact images (e.g., ∼10–40 frames) of the same sample. These raw images are then combined to create a higher resolution final contact image, visualized through the smartphone screen (Reproduced with permission from [87]).

Navruz and colleagues proposed an opto-mechanical add-on which can be attached to the camera of the cellphone, as illustrated by Figure 18 [87]. The purpose of this study was to realize a portable and compact cellphone-based contact microscopy platform, or contact scope, which could image highly dense or connected samples in transmission mode with a relatively higher spatial resolution. Inside this add-on, an incoherent light source shines through the inserted sample slide directly. Then the transmitted light is collected and sampled by the top part of the glass-based tapered fiber array. As the top part of the taper has ∼9-fold higher density of fiber optic cables compared with the bottom part, the transmitted light is magnified ∼3× in each direction after the light passes through the taper, which is then focused by two parallel lenses on the camera of the cellphone. Meanwhile, a series of images needs to be captured when the taper is manually rotated with discrete angular increments of one or two degrees. By means of a customized Android application running on the smart phone, the series of images can be processed to produce the final microscopic image of the sample, which can be shown on the cellphone screen. The authors demonstrated a smart phone-based computational transmission microscope, which can image dense and connected samples with a spatial resolution of ∼1.6 μm over a field-of-view (FOV) of > 1.5 mm^2^. Moreover, the reconstruction of the microscopic image can be done directly by a customized Android application running on the smart phone and be shown on the cellphone screen.

### 4.2. Electrochemical Detection Based on Smart Phone

**Figure 19.**
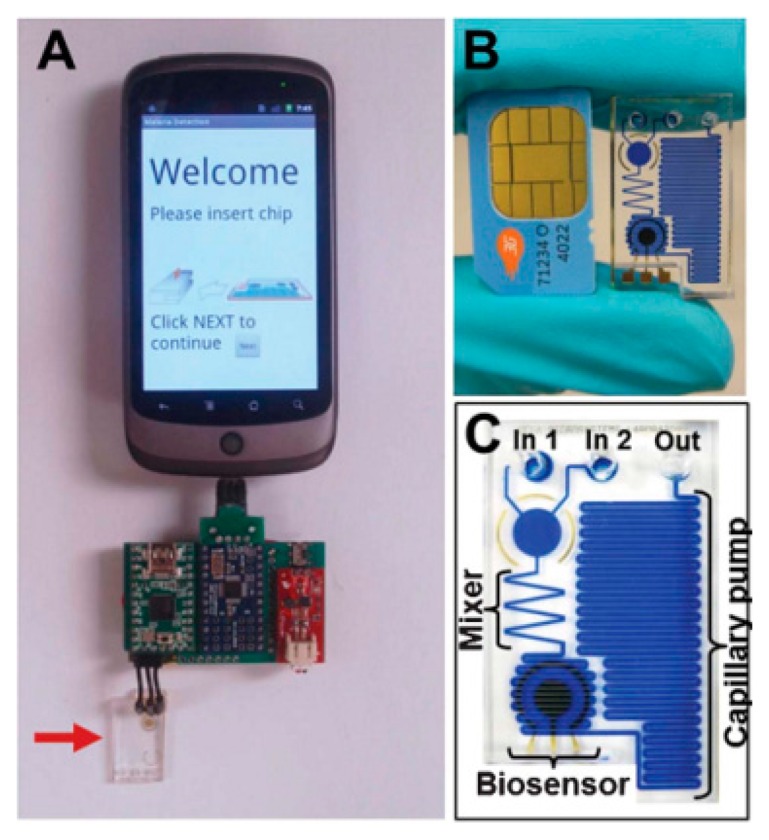
(**A**) Photograph of the assembled prototype device. (**B**) Photograph of the chip and a mobile phone SIM card for comparison. (**C**) An enlarged image of the chip with labeled components. The channels are filled with dye for improved visualization of the fluidic network (Reproduced with permission from [88]).

In order to realize a point-of-care diagnostic platform, a compact mobile phone platform integrated embedded circuit with disposable microfluidic chips was designed and tested [88]. As shown by Figure 19, a customized embedded circuit is connected to a smart phone through a universal USB connection to measure the electric signal from the biosensor on the microfluidic chip. The smart phone not only gives a user-friendly interface to guide the test but also functions as a chip reader with telecommunication capabilities. As regards the microfluidic chip part, capillary force is employed for power-free fluidic pumping. As captured antibodies are coated on the biosensors, an ELISA test takes place at the bio-sensing area, resulting in a change of the current. Therefore, if the value of the current is measured, the concentration of specific antigen in the sample can be calculated. A smart phone-based platform is presented for rapid electrochemical detection. For proof of concept, P. falciparum HRP2 antigen was detected in human serum at 16 ng·mL^−1^, which gave a five-fold greater intensity of SNR signal compared with those of irrelevant proteins.

### 4.3. Spectrometer Based on Smart Phone

**Figure 20.**
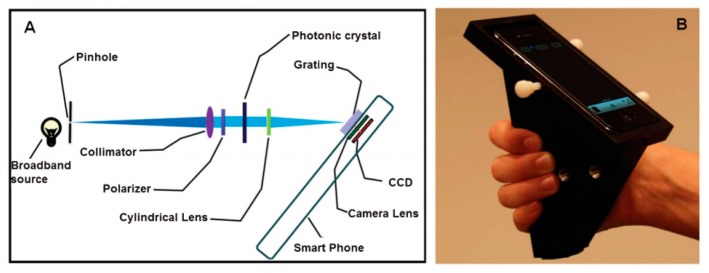
(**A**) Schematic of the optical components within the smartphone cradle. Broadband light from an external source (such as an incandescent lamp or LED) is collimated by the combination of an entrance pinhole and a collimating lens. After passing through a linear polarizing filter, light passes through the photonic crystal, which resonantly reflects one narrow band of wavelengths. The cylindrical lens increases the amount of light that is collected by the camera. (**B**) Photo of the cradle with a photonic crystal biosensor slide inserted into the detection slot (Reproduced with permission from [89]).

Apart from taking pictures, a cellphone camera can also be used as a spectrometer, a detection instrument for a label-free photonic crystal biosensor to achieve point-of-care sensing. To this end, a cradle was designed in [81] to hold the cellphone in fixed alignment, including all the optical components, to achieve precise and repeatable measurements of shifts in the resonance wavelength of biosensors during biomedical tests (Figure 20) [89]. A broadband light from the light source was filtered, collimated and linearly polarized by the pinhole, collimator and polarizer respectively before it reached the photonic crystal biosensor. As the biosensor resonantly reflected just a narrow band of wavelengths, a diffraction grating spread the remaining wavelengths to the cellphone camera to record images. In a customized Android application, the images from the cellphone's camera were converted into the biosensor's transmission spectrum in the visible wavelength range, including curve-fitting analysis with very high accuracy (0.009 nm). The biosensor was achieved by the detection of an immobilized protein monolayer and selective detection of concentration-dependent antibody binding.

## 5. Conclusions

We have described the recent advances in sensors integrated with microfluidic chips. First, SPR technology is advantageous for various reasons in developing the binding events on the sensing region with high sensitivity. Second, it is possible to detect bio-reactions in real time without a label or bio marker. In addition, integrating a microfluidic chip in the optical sensor enables effective control of small amounts of the sample, resulting in an automated high-throughput system. The approach is attractive since it is cheap and requires only brief analysis. SERS has been used for molecular detection and recognition with high sensitivity. Its sensitivity has been improved with the growth of nanotechnology as a sensing material. The main advantage of a SERS system is that the spectral signal reveals the fingerprint of specific molecules. Therefore, SERS can provide chemical and structural information of molecules without a label on the target sample.

In spite of the applicability of the SPR phenomenon to biosensing systems, it is necessary to adapt the existing microfluidic device for integration in a portable platform since many microfluidic systems need bulky and complicated operational equipment. Power consumption can be a critical issue in point-of-care clinical applications. It is clear that nanotechnology can affect the LSPR sensing system similarly to the way in which microfabrication technology enables revolution in microfluidic devices. Recent advances in nanotechnology have huge potential in terms of developing highly sensitive LSPR-based clinical devices. There is a limitation in terms of using nanohole or periodic nanoarray platforms since they require expensive fabrication techniques like electron beam lithography, and the throughput is not high. To overcome this challenge, a chemical reaction method to deposit gold nanoparticles on self-assembled monolayers would provide a possible solution. Additionally, it is worth noting that the LSPR sensing device is cost-effective and can constitute a portable commercial platform. Some of the recent literature about LSPR has reported the approach to be compatible with microfluidic platforms. The technology is promising with regard to developing a medical diagnosis system through the detection of specific molecules.

We described the miniaturized conventional optical system with maintained or improved performance which is expected to have applications mainly in on-chip point-of-care or on-chip microscopic systems. The effort of minimizing the optical image sensing is mainly focused on removing lens and overcome limitation of resolution which recently achieved better quality image over larger FOV, >20 mm^2^ with more compact and cost-efficient platform. The recent studies were including lens-less holographic microscopy which employed simple LED as light source and other techniques such as nanolense. These approaches were very sensitive which could achieve sub-100nm resolution. Another study which had been introduced is fluorescent imaging which employed microfluidic chip and illumination platform. These were able to provide real-time and very sensitive detection with simpler structure. These two approaches had proven through the demonstration that it had overcome the limitation of resolution which conventional optical microscopy had and also enabled real-time and sensitive detection by employing compact and cost-efficient platform. This allowed the future perspective of these two recent studies is providing images of micro-organism causing diseases in on-chip telemedicine and on-chip point-of-care. Current result is not available in market because the images are not still very clear for more detail observation and the size is still too large for on-chip point-of-care. Therefore more approaches are being studied for enhancing images with simpler structure of device.

For Imaging, biosensing and diagnostics based on smart phone platform, the trend for point-of-care diagnostics is the combination of consumer electronics with microfluidics. As mentioned previously, one way to achieve this goal is the optical method, which directly converts the camera of a cellphone into a lens-free microscope or a spectrometer to detect the biosignal from the add-on microfluidics part. An alternative method is the electrical method, which converts the biosignal into an electric signal without the need for optical sensing. Both approaches could utilize the increasingly powerful computational power and communication function of the cellphone. Moreover, assisted by customized software, both approaches would be user-friendly and no experience would be required to perform biochemical tests. Despite the promising advantages, there are also limitations and future developed is needed to solve those limitations: (1) Large demand for graphic process power. In order to get the high resolution image from the cellphone's camera, multiple pictures need to be taken, de-convoluted and reconstructed to form the final image. Currently this can only be done by a server computer. Thus, the cellphone cannot display real time images. (2) Cumbersome Add-ons. To realize detection by cellphones, a customized part is always needed to add on the cellphone. Integration of those parts into the cellphone is highly desired.
